# Plant-Associated Symbiotic *Burkholderia* Species Lack Hallmark Strategies Required in Mammalian Pathogenesis

**DOI:** 10.1371/journal.pone.0083779

**Published:** 2014-01-08

**Authors:** Annette A. Angus, Christina M. Agapakis, Stephanie Fong, Shailaja Yerrapragada, Paulina Estrada-de los Santos, Paul Yang, Nannie Song, Stephanie Kano, Jésus Caballero-Mellado, Sergio M. de Faria, Felix D. Dakora, George Weinstock, Ann M. Hirsch

**Affiliations:** 1 Dept. of Molecular, Cell and Developmental Biology, University of California Los Angeles, Los Angeles, California, United States of America; 2 Molecular Biology Institute, University of California Los Angeles, Los Angeles, California, United States of America; 3 Baylor College of Medicine, Houston, Texas, United States of America; 4 Departamento de Microbiología, Escuela Nacional de Ciencias Biológicas, Instituto Politécnico Nacional, Prolongación de Carpio y Plan de Ayala, Ciudad de México, Distrito Federal, México; 5 Genomic Sciences Center, National Autonomous University of México, Cuernavaca, Morelos, México; 6 Embrapa Agrobiologia, Seropédica, Rio de Janeiro, Brazil; 7 Chemistry Department, Tshwane University of Technology, Arcadia Campus, Pretoria, South Africa; 8 Dept. of Genetics, Washington Univ. School of Medicine, St. Louis, Missouri, United States of America; University Medical Center Utrecht, Netherlands

## Abstract

*Burkholderia* is a diverse and dynamic genus, containing pathogenic species as well as species that form complex interactions with plants. Pathogenic strains, such as *B. pseudomallei* and *B. mallei*, can cause serious disease in mammals, while other *Burkholderia* strains are opportunistic pathogens, infecting humans or animals with a compromised immune system. Although some of the opportunistic *Burkholderia* pathogens are known to promote plant growth and even fix nitrogen, the risk of infection to infants, the elderly, and people who are immunocompromised has not only resulted in a restriction on their use, but has also limited the application of non-pathogenic, symbiotic species, several of which nodulate legume roots or have positive effects on plant growth. However, recent phylogenetic analyses have demonstrated that *Burkholderia* species separate into distinct lineages, suggesting the possibility for safe use of certain symbiotic species in agricultural contexts. A number of environmental strains that promote plant growth or degrade xenobiotics are also included in the symbiotic lineage. Many of these species have the potential to enhance agriculture in areas where fertilizers are not readily available and may serve in the future as inocula for crops growing in soils impacted by climate change. Here we address the pathogenic potential of several of the symbiotic *Burkholderia* strains using bioinformatics and functional tests. A series of infection experiments using *Caenorhabditis elegans* and HeLa cells, as well as genomic characterization of pathogenic loci, show that the risk of opportunistic infection by symbiotic strains such as *B. tuberum* is extremely low.

## Introduction

The genus *Burkholderia* encompasses a wide range of species, including human pathogens listed as potential bioterrorist threats, opportunistic human and plant pathogens, and beneficial plant symbionts that fix nitrogen in legume root nodules. Although characteristics that benefit rhizosphere bacteria, such as ecological and nutritional flexibility, antibiotic resistance, root adherence, biofilm formation, osmotolerance, and competition for mineral nutrients may contribute to the virulence of opportunistic pathogens [1], phylogenetic evidence distinguishes the true symbionts from the opportunistic pathogens in *Burkholderia* [2], [3].

Since it was removed from *Pseudomonas* ribosomal RNA group II and moved into *Burkholderia* in 1992 [4], the genus has undergone significant taxonomic changes, with several strains transferred to *Ralstonia* as early as 1995 [5]. Approximately a decade after the original transfer of seven plant and animal pathogens to *Burkholderia*, considerable evidence began to accumulate from 16S rDNA-based phylogenetic studies indicating that this genus consisted of two distinct lineages (see references in [3]). One lineage contains species that consist of 1) mainly mammalian pathogens, e.g., the *B. pseudomallei*/*mallei* group, 2) the opportunistic pathogens, mainly the *B. cepacia* complex (BCC), 3) the plant pathogens, such as *B. glumae* and *B. gladioli*, and 4) some environmental (saprophytic) species. The second lineage includes 1) many saprophytic species that have potential for bioremediation of xenobiotics, e.g., *B. unamae* and *B. xenovorans*, 2) species that live in symbiotic relationships with plants, either as nitrogen-fixing inhabitants of legume nodules, such as *B. tuberum* and *B. phymatum*, and 3) plant growth-promoting bacteria (PGPB), for example, *B. phytofirmans*.

Trees based on other genes such as *acdS* [6] or *recA* [7] also supported the separation into two distinct clades. Perin et al. [8] not only obtained a separation into two lineages based on 16S RNA phylogeny, but also found that genes encoding two virulence factors, *cblA* (giant cable pili) and *esmR*, (BCESM, an epidemic strain marker [9]), were not detected in the clade containing the symbiotic and legume-nodulating species. By contrast, many members of the other clade possessed these gene sequences. In addition to these studies, our MLSA analysis of 77 genome sequences based on five concatenated gene sequences determined that two major groups, A and B, were delineated, based not only on phylogeny, but also on GC content [3], [10]. The B group consists of the mammalian and plant pathogens as well as the opportunistic pathogens and some environmental species, whereas the A group is divided into two subgroups, which includes many environmental and plant-associated species [3].

The goal of this report is to characterize the members of the A group more fully and propose that they be used as replacements for the opportunistic, pathogenic *Burkholderia* species, namely the BCC, which had been recommended for agricultural use or are already employed in some countries as inocula or biocontrol agents (BCA). Examples are *B. vietnamiensis*, which fixes nitrogen in association with rice roots in Asia [11], and *B. cenocepacia* and other BCC bacteria, which have been suggested for use as BCA because they kill pathogenic fungi [12]. However, major concerns about putting opportunistic pathogens into the soil have been voiced [13], [14] because some species, e.g., *B. vietnamiensis*, are common inhabitants of the lungs of cystic fibrosis (CF) patients [15], [16]. The net result is that restrictions have been placed on using BCC member species for crop use in the U.S. [17].

**Figure 1:**
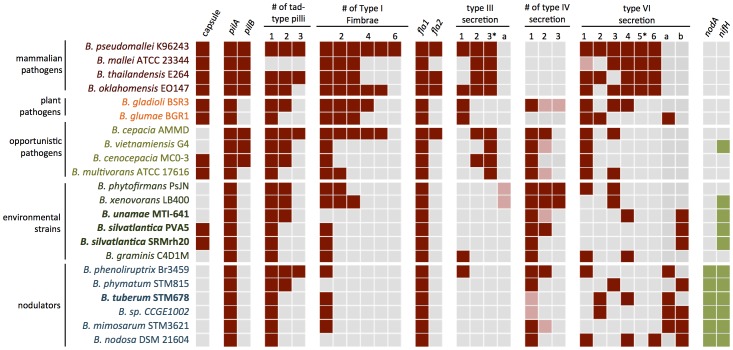
Distribution of virulence and symbiotic loci across *Burkholderia* species. Virulence-associated loci were identified using BLASTP against characteristic sequences (*B. pseudomallei* or *B. cenocepacia* ATPases for secretion systems, *B. pseudomallei* pilus genes for pilus-related clusters (red), and *B. tuberum nifH* and *nodA* sequences (green)). Non-canonical or truncated clusters are indicated in pink, and clusters highly associated with virulence for the Type 3 and Type 6 secretion systems are denoted with an asterisk.

The centers of origin of the symbiotic *Burkholderia* species are South Africa and Brazil [10]. Many southern hemisphere soils are highly acidic, arid, and low in nutrients [18]. Thus, species of the A group have significant agricultural potential for use as inocula for legume production in areas with such soils. We sequenced the genomes of four diazotrophic *Burkholderia* species in the A group, one of which nodulates legumes (Table 1). The four genomes sequenced are large, ranging from ca. 8.29 Mbp (*B. silvatlantica* SRMrh20^T^) to ca. 9.95 Mbp (*B. unamae* MTI641^T^). Here, we expand upon the analyses made by Perin et al. [8] and Estrada-de los Santos et al. [3], addressing the occurrence in these species and other members of the A clade of known virulence determinants implicated in *Burkholderia* pathogenesis [19], such as 1) motility, chemotaxis, and attachment; 2) antibiotic resistance; and 3) the type 3, 4, and 6 secretion systems (Fig. 1). We also performed functional tests to determine the pathogenic properties of the symbiotic species versus the pathogenic strains, namely by examining the extent of pathogenicity to inoculated *Caenorhabditis elegans* nematodes and HeLa cells.

**Table 1:** Bacterial strains used in this study.

Species and Strain	Phenotype	Reference or Source
*B. tuberum* STM678^T^	Forms root nodules and fixes N with siratro, bean and cowpea.	[58]
*B. unamae* MTI641^T^	Possible endophyte; capable of fixing N.	[66]
*B. silvatlantica* PVA5	Possible endophyte; capable of fixing N.	[58]
*B. silvatlantica* SRMrh20^T^	Possible endophyte; capable of fixing N.	[8]
*B. gladioli* BSR3	Plant pathogen	[67]
*B. thailandensis* E264	Pathogen	[21]
*B. vietnamiensis* G4	Nitrogen-fixing member of the BCC	[11]
*E. coli* OP50	Uracil auxotroph; food source for *C. elegans*	[22]
*Bacillus simplex* 237	Plant-growth promoting species isolated from the Negev Desert in Israel	[23]

## Materials and Methods

### Bioinformatics Analysis of Virulence Loci

Protein sequences associated with virulence in *B. pseudomallei* (for chemotaxis, attachment, Type 3 and Type 6 secretion systems; T3SS and T6SS) or *B. cenocepacia* (for the Type 4 secretion system; T4SS) were used to search the genomes of other *Burkholderia* strains using BLASTP. Top hits were identified and cluster arrangements of gene neighborhoods were verified using the Integrated Microbial Genomes tools (https://img.jgi.doe.gov/). Consensus Neighbor-Joining phylogenetic trees were constructed in MEGA 5.1 [20] using CLUSTALW alignment of several concatenated protein sequences with the p-distance method and 1000 bootstrap replicates.

### Culture of Bacteria

The bacteria used for experimental analysis are listed in Table 1. For routine culture, *Burkholderia* strains were grown on either liquid or solidified LB minus NaCl medium at 30°C, with the exception of *B. thailandensis* E264 [21], which was grown at 37°C for 48 h. *E. coli* OP50 [22] was grown on LB agar at room temperature for 24 h. *Bacillus simplex* 237 [23] grew up overnight at 30°C on LB agar. Prior to inoculation or seeding assay plates, all strains were grown in broth culture in the appropriate medium with aeration under the temperatures and for the times indicated previously. Broth cultures were adjusted to an OD_600_ = 0.1 with phosphate-buffered saline (PBS) to normalize cell numbers.

### Antibiotic Resistance Assays

The following bacteria strains were tested for antibiotic resistance: *Burkholderia tuberum* STM678^T^, *B. silvatlantica* PVA5 and SRMrh20^T^, *B. unamae* MTI641^T^, *B. vietnamiensis* G4, *B. gladioli* BSR3, and *B. thailandensis* E264 (Table 1).

In a preliminary experiment using premade antibiotic discs, we screened for antibiotics that could be used in a hole-punch filter paper experiment. The stocks for the antibiotics used for the filter paper disc assay were: ampicillin (100 µg/ml), kanamycin (30 µg/ml), chloramphenicol (25 µg/ml), gentamicin (40 µg/ml), rifampicin (15 µg/ml), streptomycin (100 µg/ml), erythromycin (15 µg/ml), nalidixic acid (25 µg/ml), and tetracycline (30 µg/ml).

From agar-grown cultures, a single colony from each plate was transferred to a 5 mL liquid culture incubated overnight at 30°C on a shaker. One milliliter of the culture was taken and centrifuged. The supernatant was removed and the bacteria were re-suspended in PBS. The solution was then diluted to an OD_600_ of 0.1. Using a cotton swab, the bacterial suspensions were spread onto agar plates. Five µL of dilutions of each antibiotic stock were added to previously autoclaved hole-punch discs, which were allowed to dry for approximately 15 min before placing them onto the bacteria-inoculated plates. The plates were incubated at 30°C until a bacterial lawn was evident, usually 1–2 days depending on the strain. Colonies were examined for zones of clearing (susceptible) or no clearing (resistant) around them [24]. A photograph was taken of each plate from the same height and at the same magnification. The radii of the clearing zones were determined by measuring on the photograph the number of pixels from the paper disc to the edge of the clearing. The pixel number was converted to millimeters using the standard conversion of 3.81 pixels per millimeter. The experiment was performed twice and a heat map summarizing the average values of five replicates for the highest concentration of the antibiotic stock was generated in R version 2.15.2.

### Culture of Mammalian Cells and Experimental Assay

Human cervical carcinoma cells (HeLa cells) were routinely cultured and passaged in T-75 flasks with Dulbecco's Modified Eagle Medium (DMEM; Gibco Life Technologies)/10% Fetal Bovine Serum (FBS; Gibco Life Technologies)/1% antibiotics (penicillin/streptomycin) and incubated at 37°C with 5% CO_2_. Cells were grown to 70–80% confluency and passaged approximately every 3–4 days and carried to a maximum of 25 passages from original stocks. Cells were washed with calcium-magnesium-free phosphate-buffered saline (CMF-PBS) prior to treatment with trypsin and before assays. For *in vitro* cytotoxicity assays, HeLa cells grown in a multi-well round-bottom plate format were washed with sterile CMF-PBS, inoculated with bacteria for an MOI (multiplicity of infection) of 50, or mock inoculated with medium alone and incubated for 4, 8, or 24 h. After the appropriate incubation period, cytotoxicity was measured via lactate dehydrogenase (LDH) release using the Cyto Tox 96 Non-Radioactive Assay kit (Promega). All work was carried out in a sterile class II laminar flow hood.

### Maintenance of *C. elegans* and Experimental Assay

*C. elegans* Bristol-N2 cultures were maintained according to methods described in Stiernagle [25]. Briefly, the worms were routinely grown on nematode growth agar medium (NGM) seeded with *E. coli* OP50, and incubated at 22°C. Nematodes from plates in which the bacterial lawn had cleared were passaged onto fresh plates with *E. coli* OP50 by aseptically transferring plugs of agar with worms on the surface. For the slow killing assays, stock plates were washed with sterile water to suspend worms and eggs, then stage-synchronized for harvesting L3-L4 larvae according to methods described earlier [25] and transferred onto NGM agar plates seeded with the bacterial strain under investigation. The initial numbers of live worms were counted and then monitored every 24 h at the same time of the day over 72 h to assess the effects of bacterial toxicity; however, some experiments continued for as long as 5 days. Death was assessed by a lack of motility and response to touch with a sterile platinum loop. All experiments were performed a minimum of three times to verify the nematode response.

For the competition experiments, the nematodes were seeded onto solidified NGM equidistant from two bacterial lawns–one comprised of *B. thailandensis* E264 and the other consisted of either the symbiotic *Burkholderia* species or *E. coli* OP50. Measurements were made as described above.

### Genomes

The four genomes sequenced for this study and their accession numbers are: *B. tuberum* STM678 (Accession Number PRJNA30619), *B. unamae* MTI641 (PRJNA59741), *B. silvatlantica* PVA5 (PRJNA51165) and *B. silvatlantica* SRMrh20 (PRJNA41353).

## Results

### Genetic/Genomic Analyses

**Flagella, Chemotaxis, and Attachment:** A number of extracellular and surface structures are involved in *B. pseudomallei* pathogenesis, including capsular polysaccharide, Type IV pili, *tad*-type pili, and Type I fimbriae [19], [26]. The *B. pseudomallei* genome contains genes encoding a full complement of such virulence factors, including the capsule [27], two Type IV pilin clusters [28], three *tad*-type pili, and six Type I fimbriae [29] (Fig. 1). The environmental and symbiotic strains contain significantly fewer such clusters, with capsule genes present in only *B. silvatlantica* SRMrh20^T^ and PVA5. All strains except the mammalian pathogens lack the Type IV pilus cluster *pilB*. The environmental and symbiotic species, except for *B. phytofirmans* and *B. xenovorans* (Fig. 1), contain only one Type I fimbriae cluster. In general, the pathogenic species have multiple clusters of Type I fimbriae. Except for *B*. *phenoliruptrix* Br3459, which has 3 of the four types, the environmental and nodulating species have either one or two types of *tad* pili (Fig. 1).

Bacterial motility is frequently associated with pathogenesis, and conflicting studies have shown that *Burkholderia* flagella are important for motility and infectivity under certain conditions. Although an early genetic study reported that interruption of the *B. pseudomallei fliC* gene with a transposon did not disrupt infection in diabetic rats or Syrian hamsters [30], a subsequent analysis showed that a *B. pseudomallei fliC* knockout was avirulent in intranasal infection of BALB/c mice [31]. More recently, a second flagellar gene cluster (*fla2*) responsible for intracellular motility was identified in *Burkholderia thailandensis* [32].

**Figure 2:**
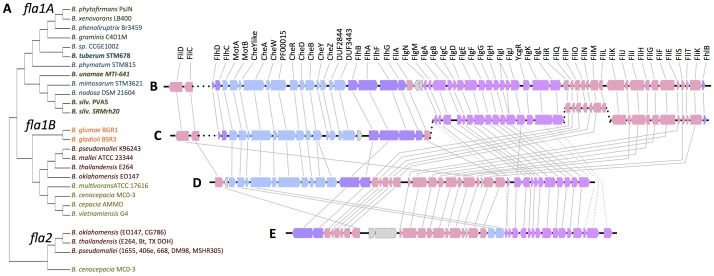
Flagellar gene clusters in *Burkholderia*. All *Burkholderia* strains share a highly similar chemotaxis and flagellar gene cluster (*fla1*) on chromosome 1. Although the A group and the pathogenic B group share high homology in gene sequence and chromosomal arrangement, a phylogenetic analysis of five concatenated protein sequences (FliC, FlgM, FlgE, FlhB, FlgJ) shows a distinct clustering of the two lineages [A]. Additionally, the A group cluster is arranged entirely sequentially [B] whereas the pathogenic B cluster is split into four different regions throughout chromosome 1 (the *B. mallei* cluster is split into 5 regions)[C]. The *fla1* gene cluster is responsible for *Burkholderia* motility on soft agar, but not for intracellular motility or plaque formation in models of infection [32]. A second flagellar gene cluster on chromosome 2 (*fla2*) is necessary for this intracellular motility. This second cluster is present only in the pathogenic strains, i.e. *B. pseudomallei, B. thailandensis*, and *B. oklahomensis* [D], and a similar cluster exists on chromosome 2 of the opportunistic pathogen *B. cenocepacia* [E].

All strains of *Burkholderia* in our analysis share a cluster of flagellar and chemotaxis-related genes with high sequence homology and high synteny (Fig. 1, Fig. 2A). In the A group of *Burkholderia* spp. (3), the chemotaxis and flagellar genes are clustered together and adjacent to one another on the chromosome (Fig. 2B). In contrast, the cluster found in members of the pathogenic clade or B group (3) is split into four different regions across the chromosome, except in *B. mallei*, where the cluster is divided into 5 regions (Fig. 2C). On the other hand, the *fla2* cluster is present only in a small percentage of strains from the *B. pseudomallei* clade, which includes *B. pseudomallei* 1655, 406e, 668, DM98, and MSHR305, *B. thailandensis* E264, Bt, and TX DOH, and *B. oklahomensis* EO147 and CG786 (Fig. 2D). Flagella have also been identified as virulence factors in the opportunistic pathogen *B. cenocepacia* [33], which has a second cluster showing divergence from the *B. pseudomallei* cluster (Fig. 2E).

**Type 3 secretion system:** The T3SS is a hallmark of pathogenic *Burkholderia* species. *B. pseudomallei* carry three such clusters on chromosome 2 (Fig. S1). The T3SS-3 locus (BPSS1529-BPSS1552) is homologous to the T3SS that modulates intracellular behavior of the human pathogens *Salmonella* and *Shigella* [19] and has been shown to be essential for endosome escape in *B. pseudomallei* infection [32]. Clusters homologous to *B. pseudomallei* T3SS-3 are present only in the *B. pseudomallei* and the *B. cepacia* groups and were not found in the plant pathogens or in any of the A group environmental or symbiotic strains (3* in Fig. 1).

Although the functions of T3SS clusters 1 and 2 in *B. pseudomallei* infection are less well-characterized [26], *B. pseudomallei* T3SS-1 (BPSS1390-BPSS1410) and T3SS-2 (BPSS1610-BPSS1629), both show homology to the T3SS of the plant pathogen *Ralstonia solanacearum*. T3SS-1 homologs are not present in *B. mallei* [34] or in the infection model *B. thailandensis* E264 [21], but homologous clusters were found in the plant pathogens and two of the A group strains, namely *B. graminis* and *B. phenoliruptrix*, a recently sequenced nodulator of *Mimosa flocculosa* [35]. A fourth type of T3SS operon dissimilar to the three found in *B. pseudomallei* is also present in the environmental strains *B. phytofirmans* [36] and *B. xenovorans* [37] (Fig. S1). This cluster is homologous to the T3SS operon in the endophytic plant pathogen *Herbaspirillum rubrisubalbicans* [38]. Mitter et al. [36] reported that the genes for the needle-forming component of the T3SS in *B. phytofirmans* PsJN appear to be missing (see Fig. S1). Genes encoding proteins for the various types of T3SS were absent in *B. tuberum*, *B. unamae*, the two *B. silvatlantica* strains, and the vast majority of the A group species (Fig. 1).

**Figure 3:**
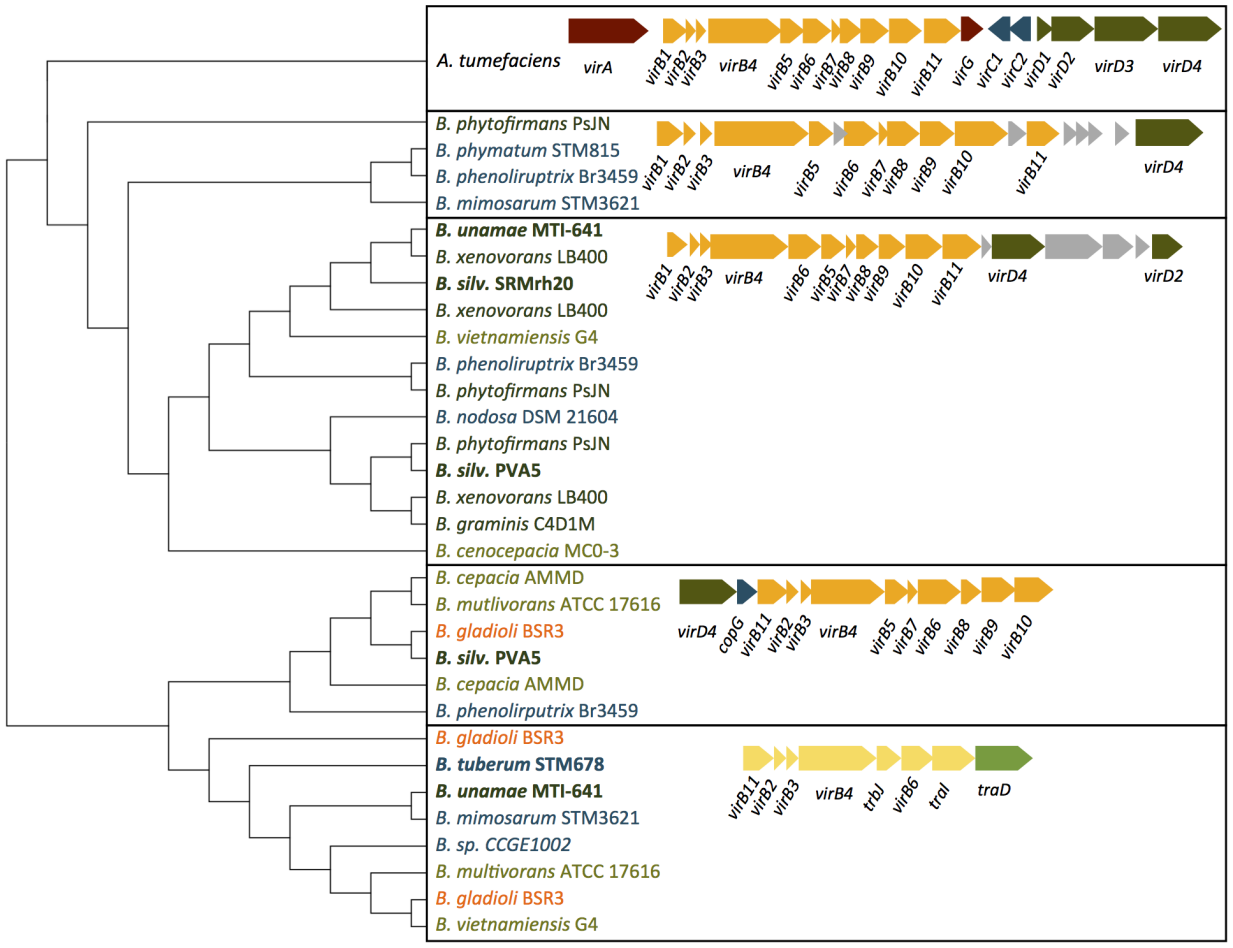
Phylogenetic analysis of Type 4 secretion system clusters in *Burkholderia* species. Three clusters with a unique gene organization have the characteristic VirB and VirD4 proteins found in the *Agrobacterium tumefaciens* cluster. A fourth, truncated cluster (yellow) is also found in many *Burkholderia* strains, including *B. tuberum* STM678^T^, but is unlikely to contribute to secretion.

**Type 4 secretion system:** The T4SS is involved in a number of functions important for pathogenesis [39], [40]. Two T4SS have been identified in *Burkholderia cenocepacia*, one chromosomally-encoded VirB/D4 type involved in plasmid mobilization, and a larger plasmid-borne cluster associated with plant tissue water-soaking infection [41]. Although neither T4SS is found in the *B. pseudomallei* group, variants are found throughout the *B. cepacia* cluster and the environmental diazotrophic *Burkholderia* strains (Fig. 1,3), including the non-pathogenic, N_2_-fixing species shown in Table 1. Only members of the BCC and the plant pathogens possess the plasmid-borne, pathogenesis-associated T4SS cluster, although variants of the standard VirB/D4 exist in up to three copies in some of the environmental *Burkholderia* genomes.

The *Burkholderia* VirB/D4 T4SS loci cluster into four types based on phylogenetic analysis of six concatenated genes, VirB1-6, each with a unique chromosomal arrangement (Fig. 3). *B. unamae* MTI641^T^, *B. silvatlantica* SRMrh20^T^, and *B. silvatlantica* PVA5 each contain clusters similar to the *B. cenocepacia* plasmid mobilization T4SS. *B. silvatlantica* PVA5 also contains a second arrangement shared with several of the opportunistic and plant pathogen strains, whereas *B. tuberum* STM678^T^ has only a conserved, but truncated and non-canonical cluster that likely is not involved in secretion (Fig. 3). None of the four symbiotic *Burkholderia* genomes that we analyzed in detail have orthologs of *virAG*, the two-component system that regulates the *vir* operon in *A. tumefaciens*.

**Figure 4:**
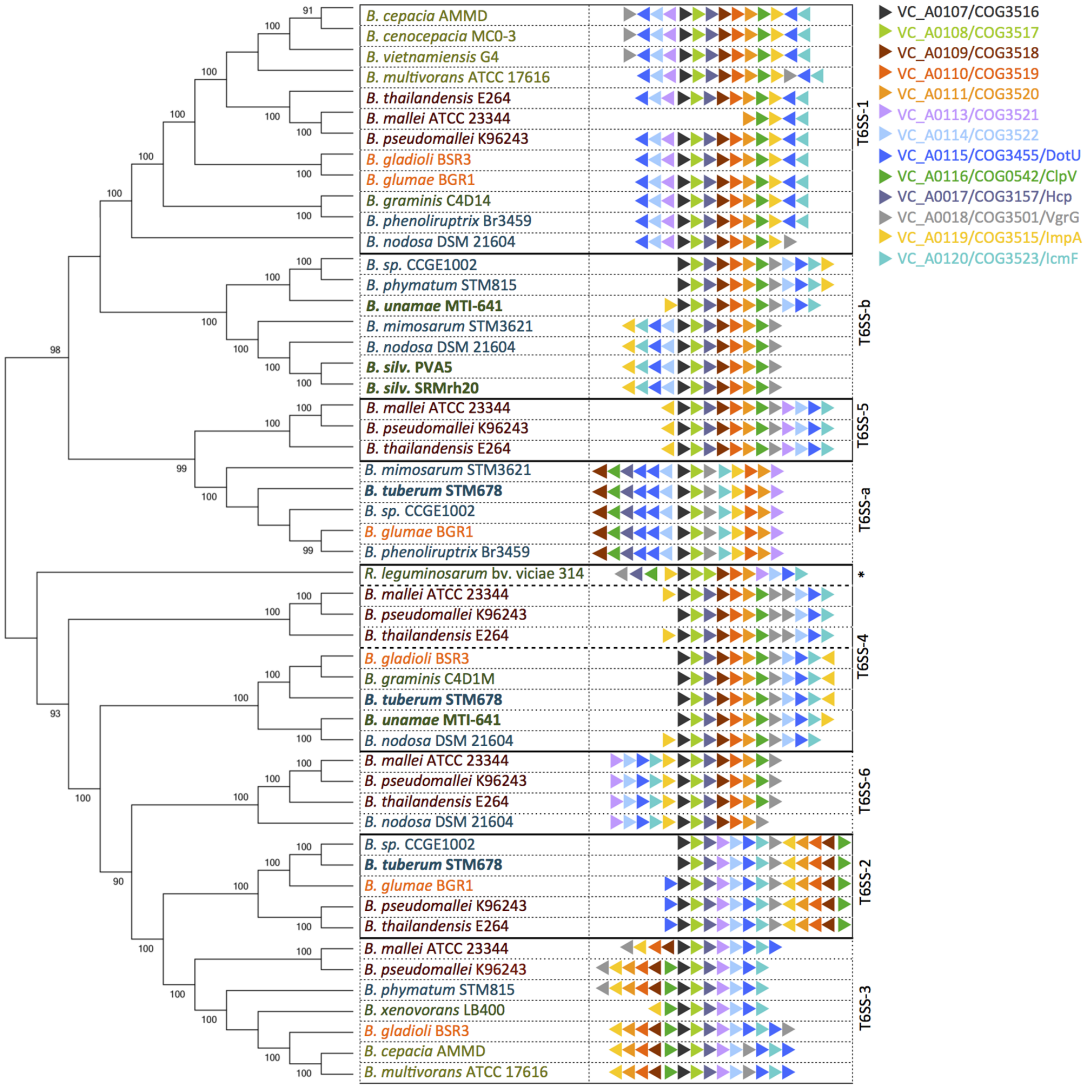
Relationship between the *Burkholderia* Type 6 secretion systems and arrangement of gene clusters. Clusters were identified with BLASTP against the *B. pseudomallei* T6SS-2, and a concatenated neighbor-joining phylogenetic tree was generated in MEGA 5.1 using highly divergent protein sequences–VgrG, Hcp, ClpV, IcmF, and the lysozyme-like protein VC_A0109. The gene arrangements of the cluster were manually verified. Although *B. oklahomensis* EO147 was found to have five T6SS clusters (Fig. 1), it was excluded from the analysis because the draft quality of the genome sequence did not allow for full reconstruction of the gene arrangement of the operon. The four symbiotic strains emphasized in this study are highlighted in bold. The *R. leguminosarum imp* region clusters with the T6SS-4 group and is indicated with an asterisk. The T6SSa and TG6SSb clusters are found only in the environmental and symbiotic *Burkholderia* species.

**Type 6 secretion system:** T6SS regulons are a commonly described feature of virulent bacterial species [42], [43]. Six different T6SS gene clusters have been identified in the *B. pseudomallei* genome [44] with distinct roles in pathogenesis and survival [45]. T6SS-5 is required for pathogenesis of *B. thailandensis*, whereas T6SS-1 has been shown to be involved in bacterial cell-cell interactions. Deletion of members of the T6SS-1 cluster leads to cells that are less able to compete with other bacterial cells in biofilms [45]. The other clusters are poorly characterized, but the *B. pseudomallei* T6SS-4 shows homology with the *R. leguminosarum imp* region (Fig. 4).

We performed a phylogenetic analysis of five concatenated protein sequences–VgrG, Hcp, ClpV, IcmF, and VC_A0109, a sequence encoding a lysozyme-like protein that is highly divergent between different strains–from the range of *Burkholderia* species spanning the pathogenic, environmental, and symbiotic clades identified in Fig. 1 (Fig. 4). All *Burkholderia* species analyzed had at least one T6SS cluster, but only *B. pseudomallei* had the full set of six. The other mammalian pathogens namely, *B. mallei, B. thailandensis*, and *B. oklahomensis* each had 5 clusters including the pathogenicity-associated T6SS-5, although T6SS-1 was truncated in *B. mallei*. The environmental and symbiotic species on average have 2 distinct T6SS clusters, but no T6SS-5 cluster (5* in Fig. 1). The phylogenetic clustering of the *Burkholderia* T6SS operons also identified two new clades with unique cluster arrangements (Fig. 4). These two clusters, here labeled T6SS-a and T6SS-b, are largely found only in the environmental and symbiotic strains. *B. tuberum* STM678^T^ has three T6SS regulons, including one with homology to *B. pseudomallei* T6SS-2 and one to T6SS-4. *B. unamae* MTI641^T^ has two clusters, one similar to T6SS-4, and the unique cluster T6SS-b, whereas *B. silvatlantica* SRMrh20^T^ and *B. silvatlantica* PVA5 each have only one cluster, the T6SS-b operon (Fig. 4).

### Antibiotic Resistance Assays

**Figure 5:**
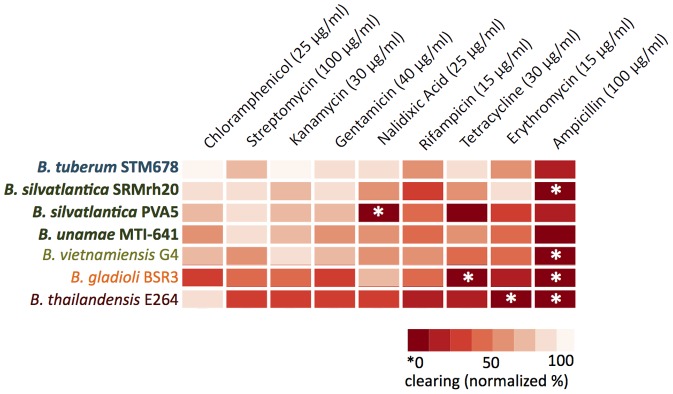
Antibiotic-resistance profiles of pathogenic and symbiotic *Burkholderia* species. Relative resistance to a panel of antibiotics for the four environmental and symbiotic strains emphasized in this study compared to the pathogenic *B. thailandensis* E264, the opportunistic pathogen *B. vietnamiensis* G4, and the plant pathogen *B. gladioli* BSR3. The average clearing of five replicate experiments at the highest antibiotic concentration are represented in the heat map. Full resistance (no clearing) is indicated with an asterisk.

Generally, soil bacteria that are opportunistic pathogens are considered to be resistant to multiple antibiotics [1], [46]. For this reason, we examined the antibiotic resistance of several plant-associated and environmental species compared to *B. thailandensis* E264, *B. vietnamiensis* G4, and *B. gladioli* BSR3. Like many pathogenic bacteria, *B. thailandensis*, exhibited varying ranges of resistance to the antibiotics tested except for chloramphenicol (25 µg/ml), to which it was significantly sensitive. *B. thailandensis* E264 was completely resistant to ampicillin (100 µg/ml) and erythromycin (15 µg/ml) with a lesser degree of resistance to most of the other antibiotics tested (Fig. 5). The two other members of the B clade [3] tested, *B. gladioli* BSR3 and *B. vietnamiensis* G4, demonstrated full resistance to ampicillin, and *B. gladioli* also exhibited complete resistance to tetracycline (30 µg/ml). Both showed varying resistance to other antibiotics, except for nalidixic acid (25 µg/ml; *B. gladioli*) and kanamycin (30 µg/ml; *B. vietnamiensis*). Of the four symbiotic strains tested, all were highly sensitive to many of the antibiotics evaluated. However, several showed resistance to ampicillin, e.g., *B. silvatlantica* SRMrh20^T^, which was fully ampicillin-resistant, whereas *B. silvatlantica* PVA5 showed resistance to nalidixic acid and tetracycline.

### Functional Assays

**Figure 6:**
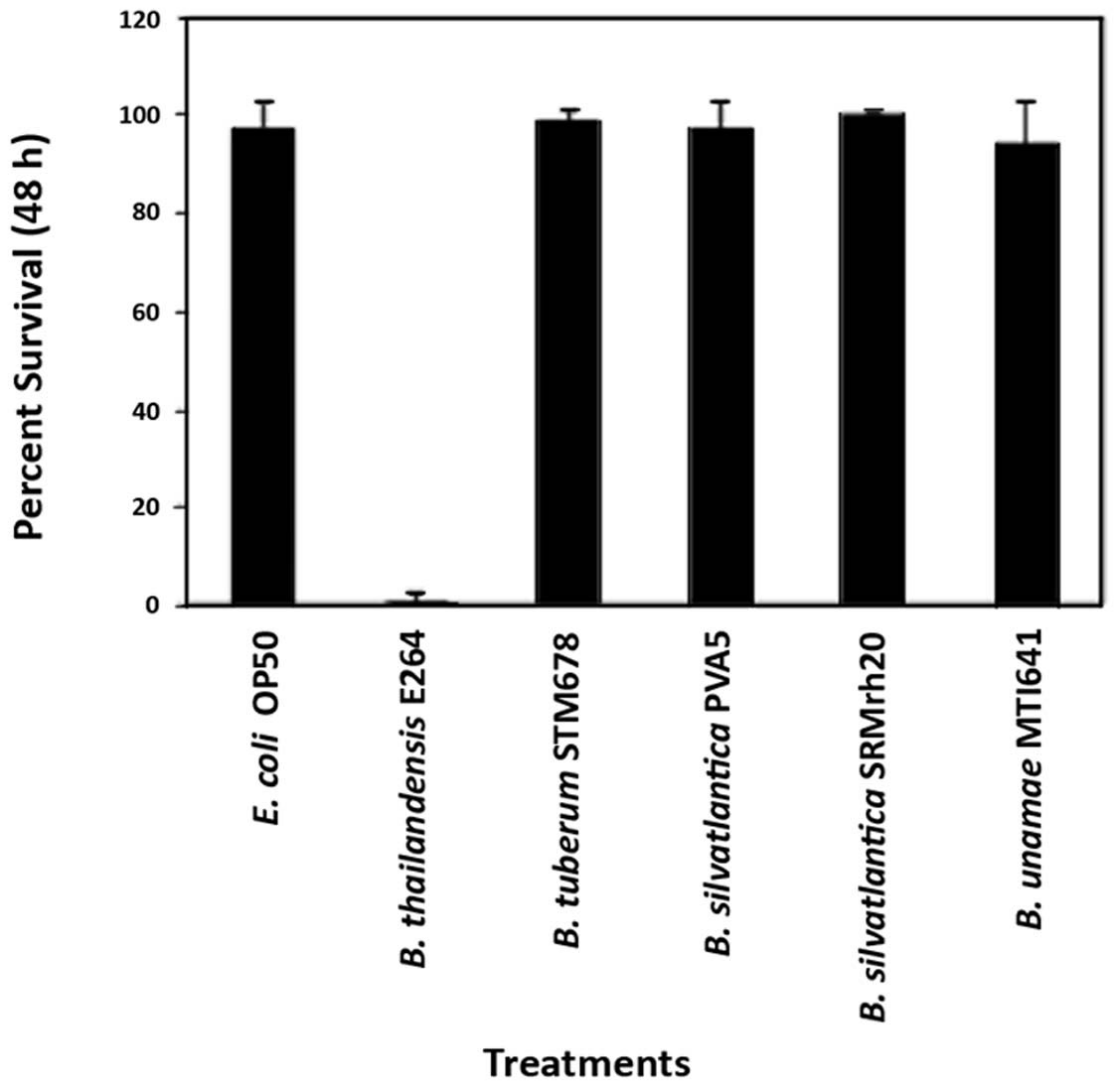
Symbiotic *Burkholderia* species are not pathogenic *in vitro* to the nematode *C. elegans* using the slow-killing assay. The edge of bacterial lawns of test *Burkholderia* strains were seeded with age-synchronized *C. elegans* N2 juvenile worms on nematode growth agar (NGM) plates. An initial count of live worms was made, and again after 24, 48, and 72 h. The percent of survivors were enumerated based on a comparison of live worms present after 72 h versus the initial count. Four symbiotic species of *Burkholderia* (*B. tuberum* STM678^T^, *B. silvatlantica* PVA5, *B. silvatlantica* SRMrh20^T^, *B. unamae* MTI641^T^) and one pathogenic species (*B. thailandensis* E264) were compared against the control, *E coli* OP50. Only the pathogenic species showed a significant and dramatic reduction in the number of live worms over the course of the experiments. All other test strains showed nearly 100% survival. Data from 48 h after the start of the experiment are shown. Error bars indicate standard error.

**Symbiotic** ***Burkholderia*** **species are not pathogenic to the nematode** ***C. elegans*** **:** Exposing the model organism *C. elegans* to bacterial strains is frequently used to assess the severity of bacterial pathogenesis [47], [48]. Using the “slow killing” assay, we compared the effects of the standard nematode food source, *E. coli* OP50 to *B. thailandensis* E264, as well as to a number of *Burkholderia* species from the A group (Fig. 6). A recently isolated plant growth-promoting bacterial (PGPB) species, *Bacillus simplex* 237, from the Negev Desert [23], had been shown to be avirulent and was used as a control (data not shown). A preliminary test using two *P. aeruginosa* stains demonstrated that nematode death was evident within 48 h (data not shown).

Bacterial pathogenesis was assessed by the percent survival of nematodes after 48 h of exposure to the bacterial lawn. When placed on lawns of the control or symbiotic strains, the nematodes were active inside the bacterial lawn. They grew and laid eggs, continuing their normal life cycle as they consumed the bacteria. In contrast, nematodes placed in the *B. thailandensis* E264 lawn remained around the periphery of the lawn and became immobile. Many nematodes died within the first 24 h in response to *B. thailandensis* E264, but a few survived up to 24 h after the start of the experiment, and all were dead after 72 h. The exact cause of death may have been due to either direct toxicity of the pathogen to the nematode or starvation from avoiding the only food provided. Further studies regarding the mechanism of pathogenesis is of interest, but beyond the scope of this study.

**Figure 7:**
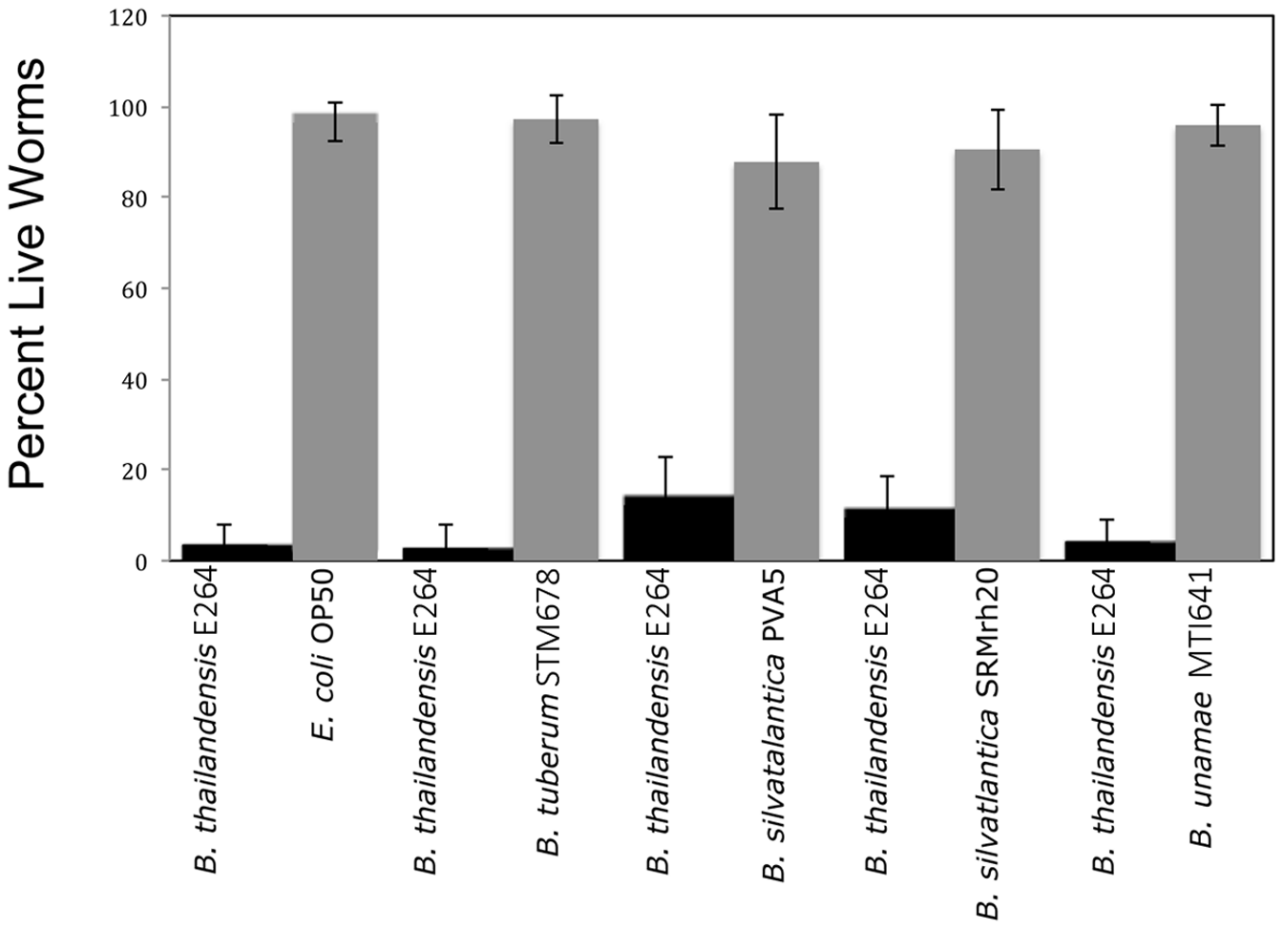
Symbiotic *Burkholderia* species are the preferred nutritional source of *C. elegans* compared to a pathogenic species. *E. coli* and symbiotic *Burkholderia* species were individually compared to *B. thailandensis* E264 to examine the response of the nematode *C. elegans* to a nutritional preference. Live worms were age-synchronized and seeded into the center of a nematode growth agar (NGM) plate equidistant from a lawn of *B. thailandensis* E264 and the aforementioned non-pathogenic strains. Counts of live worms were taken initially after seeding, and at 24, 48, and 72 h intervals. In each competition assay, the *B. thailandensis* lawn was either avoided or contained mostly dead worms, whereas live worms thrived within the lawns of *E. coli* OP50 or symbiotic *Burkholderia* species. Error bars indicate standard error.

**The symbiotic** ***Burkholderia*** **species are a preferred food source over** ***B. thailandensis*** **E264 for** ***C. elegans*** **:** Because we observed that the nematodes avoided the pathogenic *B. thailandensis* E264, we designed a “competition” experiment to determine whether chemical attractants played a role in pathogenesis. In earlier studies [49], [50], prior diet and the secretion of bacterial volatile compounds were shown to influence the choice of food for *C. elegans*. Most notably, nematodes avoid pathogenic bacteria when offered a preferred nutritional source, such as *E. coli*. If given a choice between two different bacterial lawns, the nematodes consistently avoided *B. thailandensis* E264 and chose the symbiotic *Burkholderia* species or *E. coli* (Fig. 7). Although the *B. thailandensis* E264 lawns were devoid of *C. elegans*, track marks were visible demonstrating that the nematodes explored the lawns before choosing the other food source. Within 24 h, the nematodes were active in the symbiotic *Burkholderia* lawn or surrounding agar surface. From these experiments, we concluded that the nematodes avoided the pathogenic *Burkholderia* species and were attracted to and fed upon the symbiotic species, where they were active and able to reproduce. This experiment provided evidence indicating the lack of pathogenesis in the symbiotic species towards this invertebrate model.

**Symbiotic** ***Burkholderia*** **species are not pathogenic to mammalian cells** ***in vitro*** **:** To expand our understanding of the host-microbe interactions of symbiotic *Burkholderia* species, we investigated whether the symbiotic species were toxic to mammalian cells in culture. Pathogenic bacteria invade eukaryotic cells causing cells to lyse via mechanical injury and/or toxin secretion. Either mechanism results in a release of cellular components into the surrounding environment as well as morphological changes in cell structure and attachment to neighboring cells and the culture surface.

**Figure 8:**
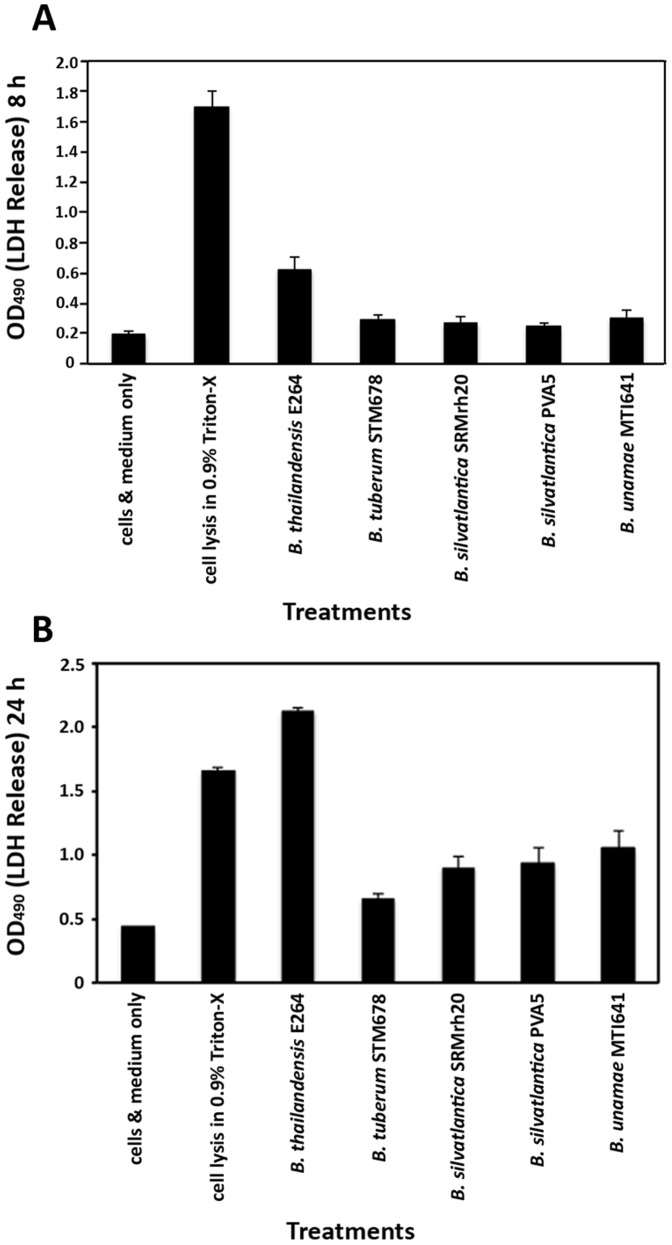
Symbiotic *Burkholderia* species are not toxic to HeLa cells in culture. Symbiotic *Burkholderia* species (*B. tuberum* STM678^T^, *B. silvatlantica* PVA5, *B. silvatlantica* SRMrh20^T^, *B. unamae* MTI641^T^) were compared to *B. thailandensis* E264 to determine if they were toxic to mammalian cells grown in culture. HeLa cells were grown until confluent and inoculated with an MOI of 50. After 8 and 24 h, samples of the supernatant of the inoculated and sham-inoculated cells were examined for LDH release using the Cyto-Tox 96 assay kit and spectrophotometric reading at 490 nm. At 8 h, only the positive control cells treated with detergent showed significant cell lysis. *B. thailandensis* E264 caused cell lysis about three times greater than that of the negative control (medium only). The effect of the symbiotic species was not statistically different from that of the negative control. At 24 h, *B. thailandensis* E264-induced morbidity had surpassed the detergent control, while the cytotoxicity caused by the symbiotic species increased only slightly from the earlier time point. Error bars indicate standard error.

To address the capacity of symbiotic *Burkholderia* to exert common pathogenic strategies on mammalian cells, we performed cytotoxicity assays on HeLa cells. After 8 or 24 h of incubation, cellular toxicity was assessed with quantification of LDH release and microscopic analysis (Fig. 8; Fig. S2). Initially (8 h post-inoculation), LDH release was two-fold greater for cells infected with *B. thailandensis* E264 compared to symbiotic *Burkholderia*, which showed levels slightly above baseline. By 24 h post-inoculation, the HeLa cells were completely cleared by *B. thailandensis* E264, at LDH release levels higher than cells treated with detergent. Microscopic observation showed that HeLa cells rounded up and did not maintain an intact monolayer. In addition, significant bacterial replication had taken place within the rounded-up cells. However, HeLa cells inoculated with symbiotic *Burkholderia* species were similar in appearance to uninfected cells (Fig. S2). Taken together, these results suggest that the symbiotic species are not harmful to mammalian cells *in vitro*.

## Discussion

The genus *Burkholderia* is a large group of bacteria composed of more than 70 species living in diverse habitats. Many of these have recently been discovered to be associated with plants either as diazotrophs or plant growth-promoting bacteria [51]. Because *Burkholderia* spp. are generally thought of as disease-causing agents, several authors have advocated that *Burkholderia* species not be used as inocula for plants [13], . Our aim in this report is to demonstrate that the two major phylogenetic groups of *Burkholderia* comprise distinct lineages, not only as previously shown by analyzing 16S rRNA and other gene phylogenies, but also by examining the *Burkholderia* genomes for virulence determinants as well as by using functional tests to determine pathogenicity.

*Burkholderia* pathogenesis is mediated by a number of virulence loci controlling different aspects of the progression of virulence from attachment, invasion, endosome escape, intracellular motility, formation of multinucleate giant cells, and cellular toxicity [19]. Many microbial virulence loci are under the control of quorum sensing (QS) systems, and AHL-mediated QS has been shown to be important in *Burkholderia pseudomallei* virulence [52], [53]. Although QS systems are widespread throughout *Burkholderia*, pathogens and opportunistic pathogens have much more complex QS systems than the symbiotic and environmental species [54]. The BraI/R QS systems among species in the A group are highly similar to each other (>75%) and cluster in a clade distinct from that of the pathogenic B group, which also has multiple QS systems.

Our bioinformatics analysis of the phylogenetic distribution of other major virulence loci across the genus *Burkholderia* shows that significant differences exist between the A and B clades (as defined by [3]). In particular, we analyzed the distribution of Type 3, 4, and 6 secretion systems across species of *Burkholderia* and found large differences across the different clades. T3SS have been shown to be necessary for pathogenesis in a number of organisms, including intracellular pathogens such as *Salmonella*, *Shigella*, and *B. pseudomallei*. Across *Burkholderia*, T3SS operons are found primarily in the true and opportunistic pathogens. Very few environmental and symbiotic strains contain T3SS operons, and of those that do, none have the T3SS-3 sequences most highly associated with virulence. For example, although *B. phytofirmans* PsJN harbors T3SS sequences, *C. elegans* worms were not affected by this strain in the slow-killing assay nor did HeLa cells lyse in response to bacterial inoculation (A.A. Angus, A. Sessitsch, and A.M. Hirsch, unpublished results). These results are consistent with the lack of the cell invasion genes in this T3SS (Fig. S1).

The T4SS is involved in a wide range of virulence-associated behaviors, including conjugative transfer of DNA, release of DNA molecules into the environment, DNA uptake and transformation, and the translocation of effector molecules to target cells. The T4SS was originally discovered in the plant pathogen Agrobacterium tumefaciens, where it transfers the T-DNA region responsible for transforming normal plant tissues into a crown gall or hairy root. The core A. tumefaciens T4SS channel is composed of 11 VirB proteins and the nucleoside triphosphatase VirD4. In *Burkholderia*, the T4SS is absent from the *B. pseudomallei* group, but was present in multiple copies in environmental strains. However, the T4SS operon in the *B. tuberum* genome was truncated and divergent in sequence from bona fide T4SS.

Although T6SS regulons are a commonly described feature from virulent bacterial species [44], one of the earliest discoveries of this assembly of genes was from the nodulating alpha-proteobacterium, *Rhizobium leguminosarum* strain RBL5523 [55], [56]. Normally, strain RBL5523 induces the formation of uninfected nodules on pea whereas it effectively nodulates vetch [55]. A Tn*5* insertion into a gene (called *impJ* for the “impaired in nitrogen fixation phenotype”) of a group of 14 genes led instead to an effective (Fix^+^) nodule phenotype on pea [55]. The effect on nodulation was temperature-dependent and correlated with the secretion of four isozymes into the supernatant of the wild-type bacterial culture after growth at a higher temperature [56]. All *Burkholderia* genomes analyzed contained at least one T6SS operon, with most having multiple clusters and *B. pseudomallei* having six. None of the environmental and symbiotic strains have the T6SS-5 cluster associated with virulence in the pathogenic *Burkholderia*, and the T6SS operons of several non-pathogenic strains also cluster into two distinct clades with different gene arrangements than the six clusters of *B. pseudomallei*.

Regarding flagella and chemotaxis, pathogenic species often have genes for two flagellar systems, one of which is employed for swimming through liquids, whereas the other is used for swarming over surfaces or within viscous environments [57]. Recently, genetic knockouts paired with photothermal nanoblade delivery of *B. thailandensis* directly into the cytoplasm of mammalian cells identified a second flagellar gene cluster on chromosome 2, which is responsible for the intracellular motility required for cell-cell spread and infection progression [32]. A second motility system was not identified in the members of the A group.

Pathogenic *Burkholderia* species are frequently resistant to multiple antibiotics, and *B. thailandensis* E264 replicates this behavior, showing resistance to a broad spectrum of antibiotics (Fig. 5). By contrast, the symbiotic and environmental members of the A group were susceptible to the vast majority of antibiotics tested. In addition to being susceptible to most antibiotics, we earlier showed that *B. tuberum* was incapable of growing at 37°C and 40°C [58] when plated during the early growth phases. These are temperatures at which pathogenic microbes frequently grow, but nonpathogenic species do not [59]. Although stationary phase *B. tuberum* cultures were more tolerant of higher temperatures (unpublished), the bacterial cells took 4 days to recover from the high temperatures due to their slow growth. These results strongly suggest that the symbiotic species are not adapted to living at mammalian temperatures.

*C. elegans* has been employed in a number of earlier investigations to be an excellent model for testing whether similar or even identical functions are responsible for virulence in humans [47], [48]. Our functional analysis demonstrated that the four symbiotic strains highlighted in this study did not kill *C. elegans*. In contrast, *B. thailandensis* E264 was very effective in the slow-killing assay. Moreover, if given a choice between *B. thailandensis* and any of the symbiotic species for food, the nematodes always choose the latter, and reproduced under these conditions. Similarly, *B. thailandensis* E264 started to lyse HeLa cells as soon as 8 h after treatment, with the cells showing significant damage after 24 h. In contrast, the symbiotic species did not cause lysis and under the microscope, the cells incubated with bacteria from the A group were identical in appearance to uninfected cells. These results strongly indicate that the symbiotic species lack the virulence determinants that are required for killing HeLa cells.

Taken together, these data demonstrate that the symbiotic and environmental *Burkholderia* species are highly unlikely to be pathogenic to mammals based on both functional and genomic data. Furthermore, the presence of pathogenic strains in the same taxonomic genus is not an accurate predictor of the presence of virulence loci in the genomes of other members of the genus or of virulence to animal cells *in vitro*. Notably, a parallel situation exists in the more commonly used agricultural alpha-rhizobia strains (*Rhizobiaceae* and *Bradyrhizobiaceae*), yet few concerns are raised regarding the use of these bacteria as inoculants. *Rhizobium* (formerly *Agrobacterium*) *radiobacter* has been isolated from respiratory secretions of CF patients [60] and reported as causing bacteremia in cancer patients [61], [62], among other conditions (cited in the previous papers). Kuykendall et al. [63] compared the chromosomes of several bacteria from three families, *Rhizobiaceae*, *Bartonellaceae*, and *Bradyrhizobiaceae*, and found genome blocks with highly conserved genes as well as genes encoding proteins that were characteristic of either a symbiotic or parasitic lifestyle. Even though *Sinorhizobium meliloti* is the closest known relative of ‘*Candidatus* Liberibacter asiaticus’, the causative agent of citrus greening disease, little overall synteny was observed between the chromosomes of the two species. A similar result was found for all five members of the Rhizobiales although microsyntenous blocks that encode core functions were detected [63]. The bottom line is that different clusters of genes and different G+C content of genomes correlate with either the symbiotic or parasitic lifestyle in the *Rhizobiaceae* [63].

For the *Bradyrhizobiaceae*, the genus *Apifia* causes nosocomial infections and based on 16S RNA phylogeny, is related to several *Bradyrhizobium* spp. that are used as inocula for plants [64]. The provisionally named *Bradyrhizobium enterica* has recently been isolated from two patients with cord colitis and a draft genome was assembled [65]. Its closest relative based on an analysis of a subset of 400 core genes is *Bradyrhizobium japonicum* USDA 110, albeit supported by a very low bootstrap value. Moreover, no evidence yet exists to show that *Bradyrhizobium enterica* is the definitive cause of cord colitis.

The situation described for the *Rhizobiaceae* and *Bradyrhizobiaceae* resembles that of the *Burkholderiaceae* in that pathogenic species are grouped together with symbiotic and beneficial species. However, in the past, the names of the genera for the nitrogen-fixing and environmental species versus the pathogens differed in *Rhizobiaceae* and *Bradyrhizobiaceae*, but recent evidence demonstrates that *Rhizobium* and *Bradyrhizobium* also contain opportunistic human pathogens. The situation is the same for the genus *Burkholderia*, which encompasses bacteria expressing a number of diverse lifestyles. However, using MLSA [3], we could show that the B clade consists of opportunistic, plant, and mammalian pathogens as well as some environmental strains, whereas the A clade contains environmental and symbiotic species. The lack of virulence determinants in the A clade genomes studied herein as well as the differences in behavior observed between symbiotic and pathogenic *Burkholderia* species lends even more support to the separation of the A group from the B clade and the delineation of each as distinct genera. In addition, it suggests the potential for safe application of the plant-associated *Burkholderia* species in an agricultural context, especially for promoting crop growth in acidic, arid soils.

## Supporting Information

Figure S1 Gene arrangement of the three Type 3 secretion system clusters in *B. pseudomallei* K96243 and a fourth type of cluster present only in the environmental strains *B. phytofirmans* PsJN and *B. xenovorans* LB400.
(TIFF)

Figure S2 Disruption of mammalian cell integrity does not occur upon inoculation with the symbiotic *Burkholderia* species. Images of HeLa cells inoculated with pathogenic or symbiotic species were visualized at 24 h. Only cells inoculated with *B. thailandensis* E264 showed cell rounding and clumping. Cells inoculated with symbiotic species appeared similar to those that were sham-inoculated with medium only. Magnification, 1000×.
(TIFF)
