# Supplementary figures and images for: Plant-Associated Symbiotic Burkholderia Species Lack Hallmark Strategies Required in Mammalian Pathogenesis
Source: PLoS One. 2014 Jan 8;9(1):e83779. doi: 10.1371/journal.pone.0083779 (PMC3885511; doi:10.1371/journal.pone.0083779)

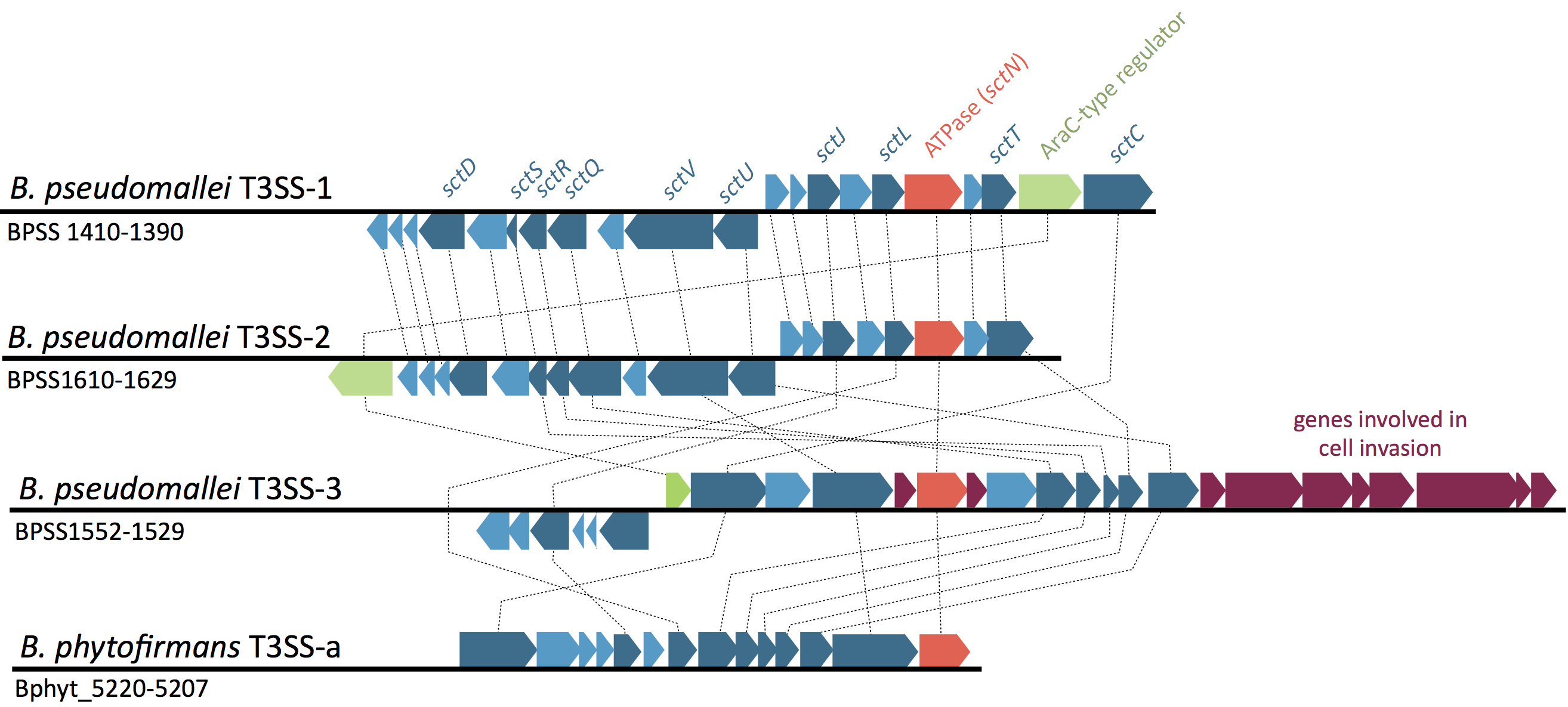

Supplement: Figure S1 — Gene arrangement of the three Type 3 secretion system clusters in B. pseudomallei K96243 and a fourth type of cluster present only in the environmental strains B. phytofirmans PsJN and B. xenovorans LB400. (TIFF) [file pone.0083779.s001.tiff]

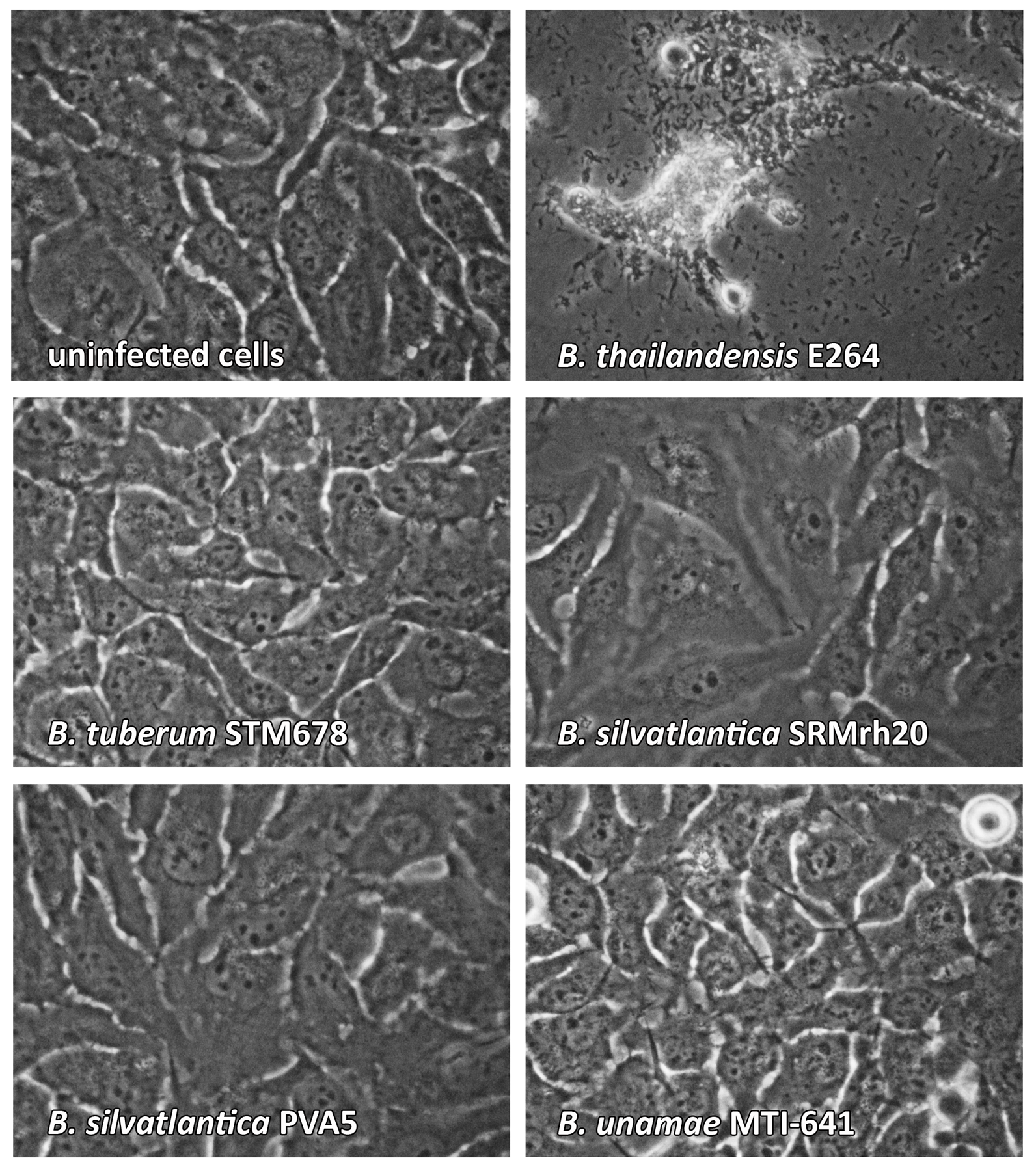

Supplement: Figure S2 — Disruption of mammalian cell integrity does not occur upon inoculation with the symbiotic Burkholderia species. Images of HeLa cells inoculated with pathogenic or symbiotic species were visualized at 24 h. Only cells inoculated with B. thailandensis E264 showed cell rounding and clumping. Cells inoculated with symbiotic species appeared similar to those that were sham-inoculated with medium only. Magnification, 1000×. (TIFF) [file pone.0083779.s002.tiff]
